# The role of discrimination and adverse childhood experiences in disordered eating

**DOI:** 10.1186/s40337-023-00753-8

**Published:** 2023-02-27

**Authors:** Jillian D. Nelson, Laura N. Martin, Alyssa Izquierdo, Olga Kornienko, Alison E. Cuellar, Lawrence J. Cheskin, Sarah Fischer

**Affiliations:** 1 Department of Psychology, George Mason University, 4400 University Drive, Fairfax, VA 22030 USA; 2 Department of Health Administration and Policy, George Mason University, 4400 University Drive, Fairfax, VA 22030 USA; 3 Department of Nutrition and Food Studies, George Mason University, 4400 University Drive, Fairfax, VA 22030 USA

**Keywords:** Perceived discrimination, Adverse childhood experiences, Eating disorder pathology, Disordered eating

## Abstract

**Background:**

In clinical research, there has been a call to move beyond individual psychosocial factors towards identifying cultural and social factors that inform mental health. Similar calls have been made in the eating disorders (ED) field underscoring the need to understand larger sociocultural influences on EDs. Discrimination is a social stressor that may influence mental health in similar ways to traumatic or adverse childhood experiences (ACEs). Given the high rates of EDs and discrimination among marginalized groups, it is vital to understand the role of discrimination and ACEs as predictors of ED symptoms in these populations. The aim of this study is to examine how perceived discrimination predicts ED pathology when statistically adjusting for gender, race, and ACEs.

**Methods:**

The diverse study sample consisted of 331 undergraduate students from a longitudinal cohort study (ages 18–24; 66% female; 35% White/non-Hispanic). Participants completed measures of everyday discrimination, ACEs, and ED pathology.

**Results:**

Following adjustment for multiple statistical comparisons, the frequency of daily discrimination predicted all ED symptoms above and beyond history of ACEs. In follow-up analyses, number of reasons for discrimination predicted cognitive restraint and purging. Differences in ED symptomatology were found based on the reason for discrimination, gender, and race. Specifically, those who experienced weight discrimination endorsed higher scores on all ED symptoms, and those experiencing gender discrimination endorsed higher body dissatisfaction, cognitive restraint, and restriction. People of color endorsed higher restriction, while female participants endorsed higher scores on all ED symptom with the exception of cognitive restraint.

**Conclusion:**

Discrimination is a salient risk factor for ED symptoms even when accounting for individuals’ history of ACEs. Future research should utilize an intersectional approach to examine how perceived discrimination affects ED pathology over time. (Word count: 234).

## Plain English summary

Adverse childhood experiences (ACEs) increase risk for eating disorders (EDs). Discrimination based on race, gender, and gender and sexual identity is also linked to ED behaviors. This paper examined whether discrimination impacted ED behaviors when ACEs were considered to understand how they both might play a role in risk for EDs. Findings suggest that experiences of discrimination may have a greater impact on eating disorder symptoms in college students than a history of ACEs. More research is needed to understand the negative impacts of discrimination on eating disorders, in addition to history of trauma. Clinicians should attend to the ways discrimination may impact their clients’ eating disorder behaviors, and whether individuals experience bias or discrimination when seeking eating disorder treatment.

## Introduction

Researchers and clinicians have largely focused on identifying and mitigating individual psychosocial factors contributing to the development and maintenance of eating disorders (EDs). Examining cultural and structural risk factors has been called for in psychological research [1]. Levine [2] and others have long described the impact of broader sociocultural factors on risk for the development of EDs. For example, they have described how Western culture emphasizes heteronormative ideals related to appearance, placing pressure on young women to be sexually appealing to heterosexual men [2, 3]. This theory acknowledges that aspects of gender and weight discrimination are related to the development and maintenance of EDs, while more recent research has examined the impacts of everyday experiences of discrimination [4]. Studying the impacts of discrimination at the individual level is one step towards understanding the effects of cultural and structural factors that impact individuals differently based on their identities.

Experiences of discrimination are associated with negative mental and physical health outcomes [5–9], and ED symptoms specifically [4]. Ethnic/racial, sexual, and gender minority groups all have higher rates of EDs when compared to their non-minoritized counterparts [10, 11]. Studies have found that racial discrimination [12, 13], gender and sexual discrimination [4], and weight-based discrimination [14] are related to higher ED pathology among marginalized groups.

The impact of discrimination on EDs may be best understood within the context of adverse childhood experiences (ACEs) due to the established impacts of trauma on EDs. ACEs include experiences of childhood maltreatment or trauma such as neglect, and emotional, physical, and sexual abuse [15]. A large body of empirical work has established a link between certain ACEs and EDs. Most individuals with EDs report a history of ACEs [16] and childhood maltreatment is a non-specific risk factor for EDs [17]. Individuals with an ED and ACE history tend to have more severe ED symptoms, including higher food restriction and greater concern about weight and shape, greater comorbid psychopathology, and worse treatment outcomes [18–21].

Adults with a history of ACEs are also more likely to report instances of lifetime discrimination [5]. A history of ACEs may amplify how one is impacted by discrimination later in life. Previous research found that individuals who experienced four or more ACEs, experienced greater mental health symptoms in the presence of discrimination than those who experienced two or fewer ACEs [22]. To fully understand the impacts of discrimination, another potentially traumatic interpersonal stressor, on ED pathology, it should be studied within the context of ACEs.

There is growing recognition that experiences of discrimination can impact individuals in a similar manner to traumatic experiences or ACEs [e.g., 9] via shared mechanisms. ACEs may lead to emotion regulation deficits that put individuals at risk of psychological distress [23] and disordered eating can serve as a coping mechanism [23–25]. Similarly, discrimination may elicit a psychological stress response that can result in negative emotions (e.g. anxiety [13, 26]). ACEs and discrimination may also be linked to ED pathology through maladaptive beliefs about oneself or one’s body. Internalized views of oneself, self-criticism, and low self-esteem have been shown to mediate the relationship between ACEs and eating pathology [27–31]. In line with these studies, discrimination has been shown to influence negative internalized beliefs toward oneself [32–34]. Because ACEs and discrimination may engender ED symptoms via similar pathways, it is likely that experiencing a greater number of ACEs and greater frequency of daily discrimination would lead to greater cumulative ED pathology.

Certain characteristics of discrimination may make it a more salient interpersonal stressor than ACEs, uniquely impacting ED behaviors. Discrimination can occur throughout the lifetime, while ACEs, by definition, occur during childhood. Discrimination may be unavoidable as it is often a chronic, daily stressor that affects members of marginalized groups who are embedded in societies characterized by bias, prejudice, and inequality [5, 35]. Individuals who experience this pervasive stressor may perceive these events as an inevitable threat to their well-being, which can result in trauma-related stress symptoms and poor health outcomes [35–37]. While ACEs are commonly assessed in healthcare settings, historically, clinicians and researchers have *not* gathered information on recently experienced discrimination when assessing potentially traumatic events. For these reasons, more attention must be paid to discrimination, and it is important to understand whether discrimination is associated with ED behaviors above and beyond the documented effects of ACEs.

The current study investigates the impact of ACEs and perceived discrimination on ED symptoms in an ethnically and racially diverse undergraduate sample. We sought to examine the association of both factors in the same analysis for several reasons. First, few studies examine the impact of discrimination on EDs, thus more research is needed on this issue. Second, there is a lack of research on the cumulative impact of ACEs and discrimination on EDs despite associations between ACEs and discrimination, and a high prevalence of ACEs in individuals with EDs. Finally, there are important differences in the way discrimination is experienced that may make it a salient risk factor for ED symptoms (i.e., recency, chronicity, uncontrollability, social identity threat). Therefore, we sought to understand whether total discrimination (a measure of the frequency of everyday experiences of discrimination) is associated with ED symptoms above and beyond the effects of ACEs. We also examine whether ACEs moderate the relationship between discrimination and ED symptoms. ED behaviors and cognitions were examined separately (e.g. restriction, binge eating, body dissatisfaction) to determine whether associations were unique to specific ED symptoms. We hypothesized that total discrimination would account for unique variance in all ED symptoms over and above ACEs. We also hypothesize a synergistic interaction between discrimination and ACEs on ED symptoms.

Further, we conducted exploratory analyses to understand the role of different identities (i.e., gender, race) and perceived reasons for discrimination in the prevalence of ED symptoms. We examined differences in ED pathology and total discrimination across gender and race and differences in ED symptoms based on which aspect of one’s identity individuals perceived to be the target of discrimination, including their race, gender, and weight. A meta-analysis by Mason and colleagues [4] found significant associations between ED symptoms and race, gender, and weight discrimination, so we hypothesized that individuals endorsing these forms of discrimination would endorse greater ED pathology. Finally, to explore how holding intersecting identities that may be targets of discrimination [38] could be disproportionately linked to ED pathology, we conducted additional regression analyses to investigate the association between the number of reasons for discrimination and ED pathology. We hypothesized individuals who endorsed more reasons for discrimination would endorse greater ED pathology.

## Method

### Participants

Participants were first-time freshman undergraduate students at a large Mid-Atlantic university who enrolled in a longitudinal cohort study [39]. The initial sample consisted of 381 participants. Due to incomplete data, 50 participants were excluded from the current analyses, leaving a final sample of 331. Participants ranged in age from 18 to 23 years (*M* = 18.54, *SD* = 1.17). Sixty-six percent of the sample identified as female and 3 participants identified as a gender other than male or female. The sample was a majority non-white. Thirty-five percent of the sample identified as White, 25.7% Asian American/Pacific Islander, 12.7% Latinx, 12.1% Black or African American/non-Hispanic or Latinx, 9.1% two or more races, 1.5% Black or African American/Hispanic or Latinx, and 3.9% identified as another race/ethnicity. Regarding sexual identity, 78.9% of the sample self-identified as straight, 12.4% as bisexual, 3.6% as gay or lesbian, 3.9% as unsure, and 0.6% of as “something else”. We chose to use the baseline assessment of the cohort data as it represented the first semester of college for the emerging adult participants. We wanted to examine the nature of the relationship between ACEs, discrimination, and ED symptoms because participants were transitioning into young adulthood, and often out of their childhood environments. Thus, by definition, they were not currently experiencing adverse childhood events, but were potentially experiencing discrimination as they transitioned to new settings and roles.

### Measures

#### Adverse childhood events (ACEs)

ACEs were assessed using the ACEs-10, a 10-item measure adapted from the CDC-Kaiser Permanente Ace Study [15], assessing maltreatment and other adverse experiences before the age of 18. To remain consistent with the typical ACEs measure, dichotomous variables were created with a score of 0 (‘No, has not happened to me’) or 1 (‘Yes, has happened to me’). Due to experimenter error, one item was entered incorrectly, capturing personal experience of physical violence rather than witnessing intimate partner violence. Since another item already assesses experiencing physical abuse, this item was removed for a total of 9 items. We expect minimal effect of the missing item because research has demostrated that witnessing intimate partner violence is only weakly associated with EDs versus other ACEs items (e.g., emotional negelct, physical abuse, sexual abuse). A study examining ACEs in adults with EDs found individuals with EDs actually were significantly less likely to report witnessing intimate partner violence on the ACEs checklist than a nationally representative sample of adults [40]. In a clinical ED sample, witnessing intimate partner violence was one of the least likely ACEs to be endorsed, with only physical neglect and having a family member in prison being less likely [41]. Yet another study found that emotional neglect, physical abuse, and sexual abuse, but not witnessing intimate partner violence, predicted binge eating disorder [42]. In our sample, total scores ranged from 0 to 8 (*M* = 1.57, *SD* = 1.77), with higher total scores indicating greater number of ACEs. In order to be consistent with our reporting of other measures, we have calculated internal consistency for the nine included items of the ACEs checklist. The Cronbach's alpha was α = .671. Exposure to traumatic events is not considered a latent psychological construct [43]. It is instead measured as a checklist of events, which may or may not be related to each other. Therefore, we would not necessarily expect high internal consistency between items of the ACEs checklist.

#### Everyday discrimination scale (EDS)

The EDS [44] was used to measure lifetime experiences of and perceived reasons for discrimination. The 5-item scale asks participants to indicate the frequency that unfair treatment in interpersonal experiences occur (e.g., “You receive poorer service than other people at restaurants or stores”). Responses are rated on a scale from 0 (‘Never’) to 5 (‘Almost every day’). Participants are then prompted to attribute the reason for these experiences and are allowed to select as many options as apply, including gender, race, and religion, among others. In the current study, total discrimination was scored by creating a sum score of the 5-items assessing frequency of everyday discrimination. Number of reasons for discrimination was calculated by summing the total number of reasons endorsed as a target of discrimination. The measure had acceptable internal consistency (α = .75).

#### Eating pathology symptoms inventory (EPSI)

The EPSI [45] is a multidimensional 45-item self-report measure of eating pathology assessing eight factors: Body Dissatisfaction, Binge Eating, Cognitive Restraint, Excessive Exercise, Restricting, Purging, Muscle Building, and Negative Attitudes Toward Obesity. Participants are prompted to rate items based on frequency over the past four weeks on a scale from 0 (‘Never’) to 4 (‘Very Often’); higher scores indicate greater ED severity. Due to space constraints in the parent study, the 31 items measuring Body Dissatisfaction, Binge Eating, Cognitive Restraint (“I tried to exclude “unhealthy” foods from my diet”), Restricting (“People would be surprised if they knew how little I ate”), and Purging were included. Since the EPSI was developed to measure dimensions of ED behavior, the five factor scores were used separately. Internal consistency of scales in the current study ranged from αs = .75–.89.

### Data collection and analysis

#### Procedure

The current study is a secondary data analysis from a larger parent study examining health, health behaviors, and mental health as predictors of college completion and the influence of individual factors on student mental health, physical health, and wellbeing [39]. Participants were recruited through flyers on campus, brief in-class presentations, online video, postcards distributed in class, and email. Participants completed an online survey measuring physical and emotional health, nutrition, sleep, civic engagement, and academic success. They also completed an in-person visit at the University public health clinic where they were asked their medical history and underwent a physical exam and blood tests. Participants are asked to participate in the study for four years, completing the online survey once per semester and the in-person visit each fall semester. Study procedures were approved by the George Mason University Institutional Review Board.

#### Data analysis

Participants who did not complete an entire measure in the current analysis were excluded from the study sample. Participants missing less than 5% of data from study measures were retained and missing data were replaced per standard procedures [46]. Notably, for all item-level missing data replaced, less than 1% of data were missing (1–3 participants per item). Data on the EPSI and EDS were replaced using median imputation, and missing data on the ACEs and EDS dichotomous yes/no variables were replaced with 0. All statistical analyses were conducted using IBM SPSS Statistics (Version 27). Descriptive statistics were calculated to characterize the sample on demographics and key variables. Bivariate correlations of ACEs, discrimination, and ED scales were also calculated. To test primary study aims, five hierarchical multiple regressions were run with each EPSI subscale as the dependent variable. In step 1, gender and race were entered to control for the impact of demographic factors known to be associated with ED symptoms [47]. In step 2, the total ACEs score was entered. In step 3, total discrimination was entered to examine whether discrimination played a role in ED symptoms above and beyond total number of ACEs experienced. The final step of the model included the interaction term between centered total discrimination and ACEs (to avoid multi-collinearity and improve interpretability). A significance level of α = .05 was applied for testing all study hypotheses. Since five multiple hierarchical regressions were run on the same sample, results were also examined after adjusting for multiple comparisons using Bonferonni’s correction (α = .01).

For follow-up analyses, we ran independent samples t-tests to understand differences in study variables based on aspects of identity (i.e., gender, race) and forms of discrimination (i.e., gender, race, weight). We ran additional regression analyses including the number of reasons for discrimination on ED symptoms. In step 1 of the regressions we controlled for gender, race, and ACEs. In step 2, we entered total discrimination and number of reasons for discrimination.

## Results

### Sample characteristics

Mean scores of the five ED subscales are listed in Table 1. Approximately 62% of participants reported having experienced at least one ACE before age 18, and 16% reported experiencing 4 or more ACEs (*M* = 1.602, *SD* = 1.78). The most reported ACEs were emotional abuse and emotional neglect (see Table 2). Eighty-three percent of participants reported experiencing discrimination (*M* = 4.77, *SD* = 4.00). The most frequently reported reasons for experiences of discrimination were gender, race, and age (see Table 2). Weight as a reason for discrimination was endorsed by 37 participants and discrimination due to sexual orientation/identity was endorsed by 18 participants. Bivariate correlations between key variables were calculated and are reported in Table 3.

**Table 1:** Sample scores for key variables

Variable	*M* (SD)
Body dissatisfaction	10.56 (6.94)
Cognitive restraint	4.05 (2.86)
Binge eating	8.05 (6.34)
Restriction	6.04 (5.59)
Purging	1.02 (2.50)
ACEs	1.57 (1.77)
Discrimination	4.77 (4.01)

Eating disorder subscale scores measured by the *Eating Pathology Symptom Inventory* (*EPSI*); ACEs = total number of adverse events endorsed on the *Adverse Childhood Events scale* (*ACEs*); Discrimination = total frequency score as measured by *Everyday Discrimination Scale* (*EDS*)

**Table 2:** Endorsement of Adverse Childhood Events and discrimination

	n (%)
Total # of ACEs	
0	126 (38.1)
1	66 (19.9)
2	53 (16.0)
3	33 (10.0)
4 or more	53 (16.0)
ACEs type	
Emotional abuse	109 (34.7)
Emotional neglect	104 (31.9)
Family member mental illness	87 (26.4)
Loss of biological parent	69 (21.0)
Family member substance use	56 (17.0)
Physical abuse	42 (13.4)
Family member in prison	26 (7.9)
Sexual abuse	18 (5.5)
Physical neglect	17 (5.2)
Discrimination type	
Gender	152 (45.9)
Race	140 (42.3)
Age	120 (36.3)
Physical appearance	81 (24.5)
Ancestry or national origins	51 (15.4)
Height	45 (13.6)
Religion	41 (12.4)
Weight	37 (11.2)
Your education or income level	32 (9.7)
Other	35 (10.6)
Political beliefs	19 (5.7)
Sexuality	18 (5.4)
Illness	17 (5.1)

Percentage reported is the Valid Percent. Discrimination type measured by Everyday Discrimination Scale

**Table 3:** Bivariate Pearson Correlations between Key Variables

Variables	1	2	3	4	5	6	7
1. Body dissatisfaction	–						
2. Cognitive restraint	.426**	–					
3. Binge eating	.497**	.208**	–				
4. Restriction	.291**	.203**	.169**	–			
5. Purging	.548**	.382**	.379**	.274**	–		
6. ACEs	.115*	.009	.187**	.041	.044	–	
7. Total discrimination	.211**	.187**	.223**	.196**	.129*	.242**	–
8. Discrim reasons	.232**	.234**	.127**	.151**	.194**	.117*	.462**

Eating disorder subscale scores measured by the *Eating Pathology Symptom Inventory* (*EPSI*); ACEs = total number of adverse events endorsed on the *Adverse Childhood Events scale* (*ACEs*); Total Discrimination = total frequency score as measured by *Everyday Discrimination Scale* (*EDS*); Discrim Reasons = total number of reasons for discrimination endorsed on *Everyday Discrimination Scale* (*EDS*)*.* EPSI Purging, ACES, and Discrim Reasons were ln-transformed for the purpose of reporting Pearson correlation coefficients

*p < .05. **p < .01

### Hierarchical multiple regression analyses

Results of the five hierarchical moderated regressions are presented in Table 4. First, we examined how much variance was accounted for by total discrimination after statistically adjusting for race, gender, and ACEs in body dissatisfaction, binge eating, restriction, cognitive restraint, and purging. Then we examined whether there was an interaction between ACEs and total discrimination on each ED subscale. Gender accounted for a significant amount of variance in the final step of the models predicting body dissatisfaction, restriction, and purging. Women were more likely to have higher scores on these three subscales. Discrimination accounted for significant variance in the final step of the model predicting body dissatisfaction, restricting, and purging, such that higher scores on discrimination and identifying as a woman were associated with more symptoms after statistically adjusting for race, and ACES. Both ACES and discrimination accounted for significant variance in the final model of binge eating. Only discrimination remained significant in the final step of the model predicting cognitive restraint, where greater discrimination was associated with higher scores on cognitive restraint.

**Table 4:** Summary of Hierarchical Regression Analysis for Variables Predicting Eating Pathology Symptoms Inventory (EPSI) Factors

	Body dissatisfaction		Binge eating		Cognitive restraint		Restriction		Purging	
Variables	*B* (*SE*)	$\beta$	*B* (*SE*)	$\beta$	*B* (*SE*)	$\beta$	*B* (*SE*)	$\beta$	*B* (*SE*)	$\beta$
*Step 1*										
Race	−.08 (.14)	−.03	−.21 (.14)	−.09	.03 (.06)	.03	.21 (.12)	.10	.05 (.05)	.05
Gender	4.75 (.73)	.34***	1.12 (.70)	.09	.46 (.32)	.08	1.59 (.62)	.14*	.88 (.27)	.18**
*Step 2*										
Race	−.06 (.14)	−.02	−.18 (.14)	−.07	.03 (.06)	.03	.22 (.12)	.10	.05 (.05)	.05
Gender	4.88 (.72)	.35***	1.28 (.70)	.10	.46 (.32)	.08	1.63 (.62)	.15**	.90 (.28)	.18**
ACEs	.52 (.20)	.13**	.58 (.20)	.16**	−.00 (.09)	−.00	.16 (.17)	.05	.08 (.08)	.06
*Step 3*										
Race	−.07 (.14)	−.03	−.20 (.13)	−.08	.03 (.06)	.02	.21 (.12)	.09	.05 (.05)	.05
Gender	4.91 (.71)	.35***	1.30 (.68)	.10	.47 (.31)	.08	1.66 (.61)	.15**	.91 (.27)	.18**
ACEs	.33 (.20)	.09	.41 (.20)	.12*	−.08 (.09)	−.05	.01 (.18)	.00	.05 (.08)	.03
Total discrim	.35 (.09)	.20***	.32 (.09)	.20***	.14 (.04)	.20***	.28 (.08)	.20***	.07 (.04)	.11*
*Step 4*										
Race	−.06 (.14)	−.02	−.19 (.14)	−.08	.04 (.06)	.03	.21 (.12)	.10	.05 (.05)	.05
Gender	4.88 (.71)	.35***	1.27 (.69)	.10	.43 (.31)	.07	1.62 (.61)	.14**	.90 (.28)	.18**
ACEs	.36 (.21)	.09	.44 (.20)	.12*	−.05 (.09)	−.03	.03 (.18)	.01	.05 (.08)	.04
Total discrim	.37 (.09)	.21***	.33 (.09)	.21***	.17 (.04)	.23***	.30 (.08)	.21***	.07 (.04)	.11
ACEs x total discrim	−.034 (.05)	−.036	−.03 (.05)	−.04	−.05 (.02)	−.12*	−.04 (.04)	−.05	−.00 (.02)	−.01
*Model fit statistics*										
Adjusted *R*^*2*^	.16		.07		.04		.06		.03	
$\Delta$ *R*^*2*^ for Step 2	.02*		.03**		.00		.00		.00	
$\Delta$ *R*^*2*^ for step 3	.04***		.04***		.04***		.04***		.01*	
$\Delta$ *R*^*2*^ for step 4	.00		.00		.01*		.00		.00	

ACEs = total number of adverse events endorsed on the *Adverse Childhood Events scale* (*ACEs*); Total Discrim = total frequency score as measured by *Everyday Discrimination Scale* (*EDS*)

*p < .05. **p < .01. *** *p* < .001

When model results (Table 4) were examined after adjusting for multiple comparisons, ACEs were no longer a significant predictor of binge eating in the final step of the model. Discrimination was no longer a significant predictor of purging. Only gender remained significant in the final step of the model, suggesting being a woman was associated with higher purging scores. All other results remained significant. Experiences of discrimination predicted greater body dissatisfaction, binge eating, cognitive restraint, and restriction above and beyond ACEs, when statistically controlling for race and gender and adjusting for multiple comparisons.

**Fig. 1:**
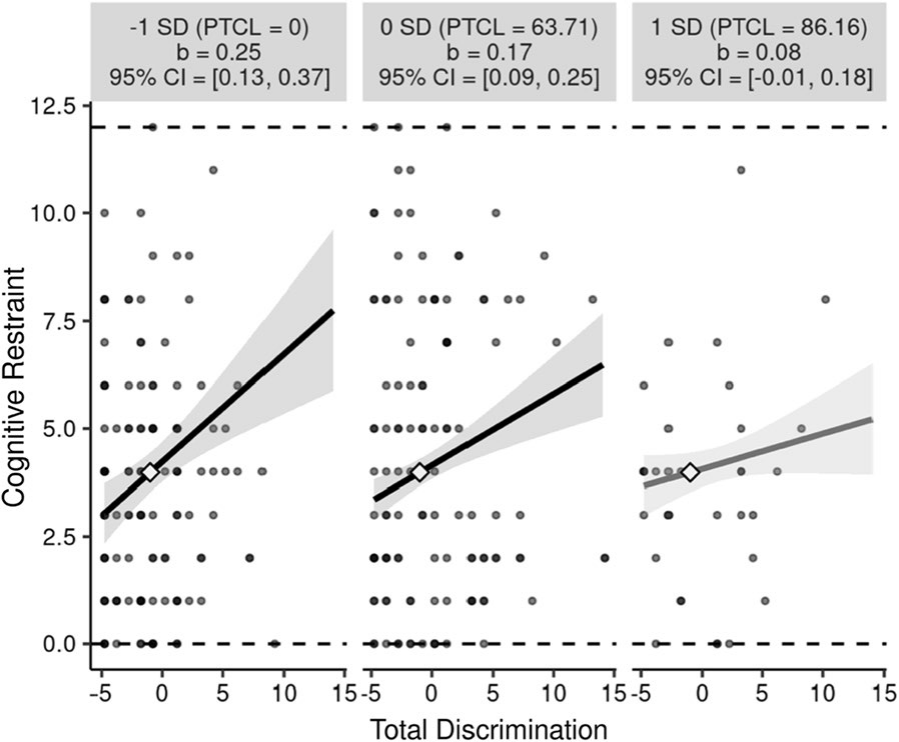
Simple Slopes between Total Discrimination and Cognitive Restraint at Levels of the Moderator (ACEs). Simple slopes significance test indicated association between discrimination and cognitive restraint was significantly different from zero at mean level of ACEs and − 1 SD. Black slope lines indicate significance; *SD* Standard deviation; *PTCL* Percentile, *CI* Confidence interval

The interaction of ACES and discrimination accounted for significant variance in the model predicting cognitive restraint. The interaction was visualized using interActive [48]. Results of the simple slopes significance test showed that the association between discrimination and cognitive restraint was significantly different from zero at mean levels of ACEs [β = 0.17, 95% CI = (0.09, 0.25)] and 1 SD below the mean [β = 0.25, 95% CI = (0.13, 0.37); see Fig. 1]. In other words, individuals with low ACEs endorsed significantly higher cognitive restraint when they reported experiencing more frequent discrimination, but significantly lower cognitive restraint with when experienced infrequent discrimination. Frequency of discrimination did not have a significant effect on cognitive restraint in those with high ACEs. No other interactions were significant.

### Follow-up analyses

Follow-up analyses were conducted to compete ACEs, discrimination, and ED symptoms across demographic variables and specific reasons for discrimination. Consistent with previous discrimination research in the field of EDs, we examined gender, race, and weight discrimination [4]. Literature suggests that sexual minorities are at higher risk for ED symptoms, potentially due to minority stress and discrimination [11]. However, in this sample, there was low endorsement of sexuality as a reason for discrimination and no follow-up analyses were run. Comparisons are summarized in Tables 5 and 6.

**Table 5:** T-test Comparison of Key Variable Scores by Gender and Race

Variable	Gender identity		Racial/Ethnic identity	
	Male (*N* = 109)	Female (*N* = 219)	White (*N* = 115)	POC (*N* = 216)
	*M* (*SD*)	*M* (*SD*)	*M* (*SD*)	*M* (*SD*)
Body dissatisfaction	6.98 (5.77)	12.34 (6.84)***	10.89 (7.33)	10.39 (6.73)
Cognitive restraint	3.73 (2.76)	4.21 (2.91)	4.05 (2.75)	4.05 (2.92)
Binge eating	7.05 (6.08)	8.58 (6.44)*	8.59 (7.28)	7.76 (5.77)
Restriction	4.95 (5.15)	6.57 (5.73)*	5.16 (5.32)	6.51 (5.68)*
Purging	0.34 (1.26)	1.37 (2.88)***	0.88 (2.65)	1.09 (2.42)
ACEs	1.82 (1.85)	1.48 (1.73)	1.54 (1.89)	1.58 (1.71)
Total discrim	5.01 (4.59)	4.63 (3.65)	3.97 (3.26)	5.20 (4.30)**

ACEs = total number of adverse events endorsed on the *Adverse Childhood Events scale* (*ACEs*); Total Discrim = total frequency score as measured by *Everyday Discrimination Scale* (*EDS*)*;* POC = people of color

*p < .05. **p < .01. *** *p* < .001

**Table 6:** T-test Comparison of EPSI Factor Scores by Discrimination Reason

Variable	Gender discrimination		Racial discrimination		Weight discrimination	
	Yes (*N* = 152)	No (*N* = 179)	Yes (*N* = 140)	No (*N* = 191)	Yes (*N* = 37)	No (*N* = 294)
	*M* (*SD*)	*M* (*SD*)	*M* (*SD*)	*M* (*SD*)	*M* (*SD*)	*M* (*SD*)
Body dissatisfaction	12.04 (6.76)***	9.31 (6.85)	10.80 (6.83)	10.39 (7.02)	15.76 (6.90)***	9.91 (6.68)
Cognitive restraint	4.49 (2.93)**	3.68 (2.74)	4.39 (2.90)	3.81 (2.81)	5.41 (3.01)**	3.88 (2.80)
Binge eating	8.22 (6.46)	7.90 (6.25)	7.94 (5.86)	8.12 (6.68)	12.05 (7.33)***	7.54 (6.03)
Restriction	6.84 (5.94)*	5.36 (5.19)	6.51 (5.30)	5.70 (5.78)	8.19 (6.24)*	5.77 (5.46)
Purging	1.30 (3.06)	0.77 (1.88)	1.21 (2.65)	0.87 (2.37)	2.70 (4.05)**	.81 (2.14)

*p < .05. **p < .01. *** *p* < .001

Results of the five hierarchical regressions which included a variable indicating the number of reasons for discrimination are displayed in Table 7. In addition to a significant effect of total discrimination, the number of reasons for discrimination accounted for unique variance in cognitive restraint and purging when controlling for gender, race, and ACEs. For the other ED subscales, total discrimination, but not number of reasons, was significant.

**Table 7:** Summary of Hierarchical Regression Analysis for Variables Predicting Eating Pathology Symptoms Inventory (EPSI) Factors

Variables	Body dissatisfaction		Binge eating		Cognitive restraint		Restriction		Purging	
	*B* (*SE*)	$\beta$	*B* (*SE*)	$\beta$	*B* (*SE*)	$\beta$	*B* (*SE*)	$\beta$	*B* (*SE*)	$\beta$
*Step 1*										
Race	−.06 (.14)	−.02	−.18 (.14)	−.07	.03 (.06)	.03	.22 (.12)	.10	.05 (.05)	.05
Gender	4.88 (.72)	.35***	1.13 (.70)	.10	.46 (.32)	.08	1.63 (.62)	.15**	.90 (.28)	.18**
ACEs	.52 (.20)	.13*	.58 (.20)	.16**	−.00 (.09)	−.00	.16 (.17)	.05	.08 (.08)	.06
*Step 2*										
Race	−.09 (.14)	−.03	−.20 (.13)	−.08	.02 (.06)	.01	.20 (.12)	.09	.04 (.05)	.04
Gender	4.71 (.71)	.34***	1.26 (.70)	.10	.35 (.31)	.06	1.55 (.61)	.14*	.82 (.28)	.16**
ACEs	.32 (.20)	.08	.41 (.20)	.11*	−.09 (.09)	−.06	−.00 (.18)	−.00	.04 (.08)	.03
Total discrim	.28 (.10)	.16**	.30 (.10)	.19**	.10 (.04)	.13*	.24 (.08)	.17**	.04 (.04)	.06
Discrim reasons	.47 (.25)	.11	.19 (.24)	.03	.31 (.11)	.17**	.26 (.22)	.07	.22 (.10)	.13*
*Model fit statistics*										
Adjusted *R*^*2*^	.17		.07		.05		.06		.05	
$\Delta$ *R*^*2*^ for Step 2	.05***		.04**		.06***		.04***		.03*	

ACEs = total number of adverse events endorsed on the *Adverse Childhood Events scale* (*ACEs*); Total Discrim = total frequency score as measured by *Everyday Discrimination Scale* (*EDS*)*;* Discrim Reasons = total number of reasons for discrimination endorsed on the *EDS*

*p < .05. **p < .01. *** *p* < .00

## Discussion

The goal of the current study was to determine whether perceived discrimination was associated with ED symptoms above and beyond history of ACEs, in an ethnically and racially diverse sample of undergraduate students. This study expanded upon previous research by taking into account ACE history, an important consideration given the strong association between ACEs and discrimination [5], and the established relationship between childhood maltreatment and EDs [17, 20, 49]. Finally, to explore the role of identity and type of perceived discrimination, ED symptomatology, and total discrimination scores were compared across groups.

Frequent discrimination was positively associated with body dissatisfaction, cognitive restraint, binge eating, and restriction above and beyond the effect of ACEs. These findings support the idea that discrimination may be a particularly salient interpersonal daily stressor with negative impacts on ED symptoms. Consistent with past research, discrimination was associated with ED behaviors that are thought to be a method of coping with negative affect (e.g., binge eating) and changing one’s appearance (e.g., restriction). It was also associated with body dissatisfaction which reflects cognitions about oneself and one’s appearance, shape or weight. Discrimination did not significantly predict purging behaviors after these statistical adjustments. This may have been due to lacking statistical power due to low endorsement of purging in the sample, and future studies should replicate these associations.

Intersectionality theory suggests that individuals who hold multiple marginalized identities (e.g., Black and female, queer male) have different experiences than individuals who only hold one marginalized identity [38]. We examined the impact of multiple marginalized identities by including the number of reasons for perceived discrimination in follow-up analyses. We found that *number of reasons for discrimination* explained unique variance in some ED symptoms (i.e., cognitive restraint and purging) when accounting for total discrimination. These findings partially support the idea that people holding multiple marginalized identities may be at greater risk for Eds. Cognitive restraint and purging may indicate attempts to change the appearance of one’s body in response to discrimination. Because discrimination is often targeted towards visible aspects of one’s identity, people may feel compelled to change aspects of their appearance that feel within their control. Future research should consider the role of multiple marginalized identities when studying the impacts of discrimination on Eds.

Inconsistent with study hypotheses, after adjusting for multiple comparisons, ACEs was not associated with any ED symptoms when discrimination was included in the model. These results are surprising given the well-documented associations between childhood maltreatment and ED symptoms [e.g., 17, 20, 49, 50]. Discrimination may have shared variance with ACEs because they contribute to ED risk via similar mechanisms. Specifically, ACEs and discrimination may both increase risk for ED through internalized negative beliefs about oneself and others [29–31, 33]. Separately, research suggests disordered eating serves as a means of coping in individuals with a history of trauma or ACEs [23–25]. ACEs and discrimination are positively associated, so it is likely that experiences of discrimination occur simultaneously with ACEs during one’s childhood, both shaping beliefs about the self or contributing to maladaptive coping behaviors.

The developmental context of our study sample is noteworthy because we focus on first-year college students, many of whom had recently left their childhood environments to start college. This life transition likely changes the impact of family dynamics that have contributed to ACEs. However, when the young adults in our sample moved away to college, the daily discrimination encounters based on their marginalized identities may followed them to their new enviropnment in college. For this reason, discrimination may actually become the more salient stressor impacting ED behaviors for our sample. In adulthood, discrimination may feel more stable and pervasive than other potentially traumatic events. It is known that attributing traumatic events as internal and stable leads to poorer self-esteem and increased hopelessness [51], and these attributions may be accurately made by people experiencing discrimination because it reflects individuals’ lived experiences. The personal and chronic nature of discrimination may engender worse self-esteem and negative affect, increasing risk for ED behaviors. Future work needs to empirically examine these mediating mechanisms as well as the role of protective factors such as ethnic-racial identity development [52] and family racial socialization (e.g., [53]) to disrupt the linkages between discrimination and ED pathology. These translational and clinical efforts to prevent and intervene in harmful effects of discrimination on health need to be guided by culturally centered and culturally adaptive perspectives (e.g., [54]).

Group comparisons allowed us to examine differences in perceived discrimination and ED pathology based on gender and racial identity as well as discrimination type (racial, gender, and weight discrimination). Individuals who perceived discrimination to be based on their gender or their weight reported greater ED symptoms, suggesting that gender and weight discrimination may be particularly salient risk factors for ED symptoms. These findings are not surprising given that ED symptoms are more commonly endorsed by females [55] and are often intended to change one’s shape or weight [56].

Interestingly, there were no differences in ED symptom endorsement for individuals who reported race discrimination versus those who did not. Previous research on the negative impacts of race-based stress and discrimination on mental health [9], includes evidence that experiencing racism predicts ED pathology [4]. Further, individuals who endorsed both race- and gender-based discrimination did not experience greater ED pathology than those who experienced only one of these forms of discrimination (race- *or* gender-discrimination). In the context of intersectionality theory, and our finding that number of reasons for discrimination predicted some ED symptoms, we may have expected that individuals who endorsed race *and* gender discrimination would have endorsed greater ED symptoms. The field would benefit from further examining the role of race-based discrimination in EDs in larger samples with greater representation across races and ethnicities.

## Conclusion

A shift to examining cultural and systemic risk factors such as discrimination and structural racism has been called for in psychological research [1]. The unique contribution of discrimination to ED symptoms in our study highlights the importance of addressing discrimination in ED populations. Discrimination may be a more salient stressor than past traumatic or adverse experiences for some individuals but have received less attention up to this point. Therefore, the field should move towards screening for experiences of discrimination in medical, mental health, and research settings. When treating individuals with EDs, it may be beneficial to explore how stress and negative affect related to past or ongoing experiences of discrimination play into ED behaviors.

It may also be important to examine the role of discrimination and bias in ED treatment. There is evidence of overweight and obesity bias among ED professionals [57], evident in the way EDs have been diagnosed until the most recent DSM-5, when atypical AN (AAN) was included as a diagnostic category [56]. Still, compared to AN, fewer individuals with AAN are being referred or admitted to treatment despite AAN being more common in many communities [58]. Additionally, men with disordered eating are less likely to be diagnosed with or seek treatment for an ED which may be due to society’s messages about who can have an ED [59–61]. If everyday experiences of discrimination have an effect on ED pathology, discriminatory practices in treatment settings may cause further harm to individuals seeking support for EDs. Clients may experience microaggressions in therapy which have been shown to negatively impact therapeutic progress, particularly if they are not addressed and processed with the clinician [62]. Clinicians should practice self-awareness and reflection to minimize client’s experience of perceived discrimination under their care.

### Limitations and future research

One limitation of the current study is that the ACEs measure was missing the item referring to witnessing domestic violence. However, based on the literature reviewed, other ACEs appear to have stronger relationships with ED symptoms. Additionally, we utilized the first wave of data from a project that follows first time college freshman over four years. Thus, our analysis is cross-sectional. We chose to cross-sectional data to establish whether or not discrimination is associated with ED symtpoms at this developmental period when adjusting for the presence of ACES.. Longitudinal studies would be particularly valuable to examine whether daily experiences of discrimination predict the onset or changes in later ED symptoms as individuals move from adoelscence to young adulthood and beyond. Ecological momentary assessment studies would allow researchers to examine the association between experiences of discrimination in daily life to ED symptoms within the same day. Future research should use these methods in order to examine the temporal and functional relationship between perceived discrimination, discrimination type, and ED symptoms.

The size of the current sample, although over 300 participants, led to limited representation of certain racial identities and did not allow for comparison by race. Although our sample was ethnically and racially diverse, the size of some subgroups represnting specific racial or ethnic categories was small. The present results need to be replicated with larger samples to examine the role of race and race-based discrimination, as well as gender and sexual identity in ED symptomatology. With substantial evidence of the relationship between discrimination and EDs, research should turn to exploring mechanisms relating discrimination and ED symptoms through mediational analyses, as well as potential protective factors, such as social support, which may buffer the effects of discrimination on ED pathology.

## Data Availability

The data that support the findings of this study are available from the corresponding author upon reasonable request.

## Abbreviations

ACEs: Adverse childhood experiences
ED: Eating disorder
EDS: Everyday discrimination scale
EPSI: Eating pathology symptoms inventory

## References

[1] Adams LM, Miller AB (2022). Mechanisms of mental-health disparities among minoritized groups: How well are the top journals in clinical psychology representing this work?. Clin Psychol Sci.

[2] Levine MP, Murnen SK (2009). “Everybody knows that mass media are/are not [pick one] a cause of eating disorders”: a critical review of evidence for a causal link between media, negative body image, and disordered eating in females. J Soc Clin Psychol.

[3] Smolak L, Murnen SK, Thompson JK (2004). A feminist approach to eating disorders. Handbook of eating disorders and obesity.

[4] Mason TB, Mozdzierz P, Wang S, Smith KE (2021). Discrimination and eating disorder psychopathology: a meta-analysis. Behav Ther.

[5] Campbell JA, Walker RJ, Garacci E, Dawson AZ, Williams JS, Egede LE (2020). Relationship between adverse childhood experiences and perceived discrimination in adulthood. J Affect Disord.

[6] Cave L, Cooper MN, Zubrick SR, Shepherd CCJ (2020). Racial discrimination and child and adolescent health in longitudinal studies: a systematic review. Soc Sci Med.

[7] Gibbons FX, Gerrard M, Fleischli ME, Simons RL, Kingsbury JH (2021). Perceived racial discrimination and healthy behavior among African Americans. Health Psychol.

[8] Pascoe EA, Smart RL (2009). Perceived discrimination and health: a meta-analytic review. Psychol Bull.

[9] Williams DR, Lawrence JA, Davis BA, Vu C (2019). Understanding how discrimination can affect health. Health Serv Res.

[10] Rodgers RF, Berry R, Franko DL (2018). Eating disorders in ethnic minorities: an update. Curr Psychiatr Rep.

[11] Nagata JM, Ganson KT, Austin SB (2020). Emerging trends in eating disorders among sexual and gender minorities. Curr Opin Psychiatr.

[12] Johnson P, Risica PM, Gans KM, Kirtania U, Kumanyika SK (2012). Association of perceived racial discrimination with eating behaviors and obesity among participants of the SisterTalk study. J Natl Black Nurses Assoc.

[13] Rodrigues YE, Fanton M, Novossat RS, Canuto R (2022). Perceived racial discrimination and eating habits: a systematic review and conceptual models. Nutr Rev.

[14] Sutin AR, Terracciano A (2013). Perceived weight discrimination and obesity. PLoS ONE.

[15] Felitti VJ, Anda RF, Nordenberg D, Williamson DF, Spitz AM, Edwards V (1998). Relationship of childhood abuse and household dysfunction to many of the leading causes of death in adults. The Adverse Childhood Experiences (ACE) study. Am J Prev Med.

[16] Mitchell KS, Mazzeo SE, Schlesinger MR, Brewerton TD, Smith BN (2012). Comorbidity of partial and subthreshold ptsd among men and women with eating disorders in the national comorbidity survey-replication study. Int J Eat Disord.

[17] Brewerton TD (2007). Eating disorders, trauma, and comorbidity: focus on PTSD. J Treat Prev.

[18] Castellini G, Lelli L, Cassioli E, Ciampi E, Zamponi F, Campone B (2018). Different outcomes, psychopathological features, and comorbidities in patients with eating disorders reporting childhood abuse: a 3-year follow-up study. Eur Eat Disord Rev.

[19] Guillaume S, Jaussent I, Maimoun L, Ryst A, Seneque M, Villain L (2016). Associations between adverse childhood experiences and clinical characteristics of eating disorders. Sci Rep.

[20] Molendijk ML, Hoek HW, Brewerton TD, Elzinga BM (2017). Childhood maltreatment and eating disorder pathology: a systematic review and dose-response meta-analysis. Psychol Med.

[21] Scharff A, Ortiz SN, Forrest LN, Smith AR (2019). Comparing the clinical presentation of eating disorder patients with and without trauma history and/or comorbid PTSD. Eat Disord.

[22] Helminen EC, Scheer JR, Edwards KM, Felver JC (2022). Adverse childhood experiences exacerbate the association between day-to-day discrimination and mental health symptomatology in undergraduate students. J Affect Disord.

[23] Hund AR, Espelage DL (2006). Childhood emotional abuse and disordered eating among undergraduate females: mediating influence of alexithymia and distress. Child Abuse Negl.

[24] Gruhn MA, Compas BE (2020). Effects of maltreatment on coping and emotion regulation in childhood and adolescence: a meta-analytic review. Child Abuse Negl.

[25] Mills P, Newman EF, Cossar J, Murray G (2015). Emotional maltreatment and disordered eating in adolescents: testing the mediating role of emotion regulation. Child Abuse Negl.

[26] Mason TB, Lewis RJ, Heron KE (2017). Indirect pathways connecting sexual orientation and weight discrimination to disordered eating among young adult lesbians. Psychol Sex Orientat Gend Divers.

[27] Anaya C, Anam S, Zickgraf HF, O’Connor SM, Wildes JE, Spalletta G, Janiri D, Piras F, Sani G (2020). Childhood trauma in eating disorders. Childhood trauma in mental disorders: a comprehensive approach.

[28] Feinson MC, Hornik-Lurie T (2016). “Not good enough:” exploring self-criticism’s role as a mediator between childhood emotional abuse & adult binge eating. Eat Behav.

[29] Groleau P, Steiger H, Bruce K, Israel M, Sycz L, Ouellette AS (2012). Childhood emotional abuse and eating symptoms in bulimic disorders: an examination of possible mediating variables. Int J Eat Disord.

[30] Jenkins PE, Meyer C, Blissett JM (2013). Childhood abuse and eating psychopathology: the mediating role of core beliefs. J Aggress Maltreat Trauma.

[31] Mitchell KS, Scioli ER, Galovski T, Belfer PL, Cooper Z (2021). Posttraumatic stress disorder and eating disorders: maintaining mechanisms and treatment targets. Eat Disord.

[32] Mason TB, Lewis RJ (2016). Minority stress, body shame, and binge eating among lesbian women: social anxiety as a linking mechanism. Psychol Women Q.

[33] Mason TB, Lewis RJ, Heron KE (2018). Disordered eating and body image concerns among sexual minority women: a systematic review and testable model. Psychol Sex Orientat Gend Divers.

[34] Watson LB, Velez BL, Brownfield J, Flores MJ (2016). Minority stress and bisexual women’s disordered eating: the role of maladaptive coping. Couns Psychol.

[35] Bombay A, Matheson K, Anisman H (2014). Appraisals of discriminatory events among adult offspring of Indian residential school survivors: the influences of identity centrality and past perceptions of discrimination. Cultur Divers Ethnic Minor Psychol.

[36] Matheson K, Foster MD, Bombay A, McQuaid RJ, Anisman H (2019). Traumatic experiences, perceived discrimination, and psychological distress among members of various socially marginalized groups. Front Psychol.

[37] Haslam SA, Jetten J, Postmes T, Haslam C (2009). Social identity, health and well-being: an emerging agenda for applied psychology. Appl Psychol.

[38] Crenshaw K (1991). Mapping the margins: intersectionality, identity politics, and violence against women of color. Stanford Law Rev.

[39] Cuellar AE, Adams LM, de Jonge L, Espina V, Espinoza L, Fischer SF (2021). Protocol for the mason: health starts Here prospective cohort study of young adult college students. BMC Public Health.

[40] Rienecke RD, Johnson C, Le Grange D, Manwaring J, Mehler PS, Duffy A (2022). Adverse childhood experiences among adults with eating disorders: comparison to a nationally representative sample and identification of trauma. J Eat Disord.

[41] Rienecke RD, Johnson C, Mehler PS, Le Grange D, Manwaring J, Duffy A (2022). Adverse childhood experiences among a treatment-seeking sample of adults with eating disorders. Eur Eat Disord Rev.

[42] Quilliot D, Brunaud L, Mathieu J, Quenot C, Sirveaux MA, Kahn JP (2019). Links between traumatic experiences in childhood or early adulthood and lifetime binge eating disorder. Psychiatr Res.

[43] Netland M (2001). Assessment of exposure to political violence and other potentially traumatizing events. A critical review. J Trauma Stress.

[44] Sternhal MJ, Slopen N, Williams DR (2011). Racial disparities in health: How much does stress really matter?. Du Bois Rev Soc Sci Res Race.

[45] Forbush KT, Wildes JE, Pollack LO, Dunbar D, Luo J, Patterson K (2013). Development and validation of the eating pathology symptoms inventory (EPSI). Psychol Assess.

[46] Scheffer J (2002). Dealing with missing data. Res Lett Inf Math Sci.

[47] Hoerr SL, Bokram R, Lugo B, Bivins T, Keast DR (2002). Risk for disordered eating relates to both gender and ethnicity for college students. J Am Coll Nutr.

[48] McCabe CJ, Kim DS, King KM (2018). Improving present practices in the visual display of interactions. Adv Methods Pract Psychol Sci.

[49] Trottier K, MacDonald DE (2017). Update on psychological trauma, other severe adverse experiences and eating disorders: state of the research and future research directions. Curr Psychiatr Rep.

[50] Caslini M, Bartoli F, Crocamo C, Dakanalis A, Clerici M, Carrà G (2016). Disentangling the association between child abuse and eating disorders: a systematic review and meta-analysis. Psychosom Med.

[51] Peterson C, Villanova P (1988). An expanded attributional style questionnaire. J Abnorm Psychol.

[52] Yip T, Wang Y, Mootoo C, Mirpuri S (2019). Moderating the association between discrimination and adjustment: a meta-analysis of ethnic/racial identity. Dev Psychol.

[53] Metzger IW, Anderson RE, Are F, Ritchwood T (2021). Healing Interpersonal and racial trauma: integrating racial socialization into trauma-focused cognitive behavioral therapy for African American youth. Child Maltreat.

[54] Anderson RE, Heard-Garris N, DeLapp RC (2022). Future directions for vaccinating children against the American endemic: treating racism as a virus. J Clin Child Adolesc Psychol.

[55] Striegel-Moore RH, Rosselli F, Perrin N, DeBar L, Wilson GT, May A (2009). Gender difference in the prevalence of eating disorder symptoms ruth. Int J Eat Disord.

[56] Diagnostic and statistical manual of mental disorders: DSM-5. 5th ed. Washington, DC: American Psychiatric Association; 2013.

[57] Puhl RM, Latner JD, King KM, Luedicke J (2014). Weight bias among professionals treating eating disorders: attitudes about treatment and perceived patient outcomes. Int J Eat Disord.

[58] Harrop EN, Mensinger JL, Moore M, Lindhorst T (2021). Restrictive eating disorders in higher weight persons: a systematic review of atypical anorexia nervosa prevalence and consecutive admission literature. Int J Eat Disord.

[59] Bohrer BK, Carroll IA, Forbush KT, Chen PY (2017). Treatment seeking for eating disorders: results from a nationally representative study. Int J Eat Disord.

[60] Coffino JA, Udo T, Grilo CM (2019). Rates of help-seeking in US adults with lifetime DSM-5 eating disorders: prevalence across diagnoses and differences by sex and ethnicity/race. Mayo Clin Proc.

[61] Strother E, Lemberg R, Stanford SC, Turberville D (2012). Eating disorders in men: underdiagnosed, undertreated, and misunderstood. Eat Disord.

[62] Owen J, Tao KW, Imel ZE, Wampold BE, Rodolfa E (2014). Addressing racial and ethnic microaggressions in therapy. Prof Psychol Res Pract.

